# Effects of a national checklist on recommended procedures among patients with type 2 diabetes

**DOI:** 10.1186/s12913-024-11940-x

**Published:** 2024-11-26

**Authors:** Øyvind Snilsberg, Tor Iversen, Anne Karen Jenum, Yuting Zhang

**Affiliations:** 1 Department of Health Management and Health Economics, University of Oslo, Oslo, Norway; 2 General Practice Research Unit (AFE), Department of General Practice, University of Oslo, Oslo, Norway; 3 Melbourne Institute: Applied Economic & Social Research, Faculty of Business and Economics, University of Melbourne, Melbourne, Australia

**Keywords:** Checklist, National quality program, Physician incentives, Diabetes management, Norwegian Diabetes Register for Adults

## Abstract

**Background:**

Type 2 diabetes (T2D) is a common, potentially disabling, and costly chronic condition that requires consistent management. In 2008, Norway introduced a national checklist outlining services to include in an annual T2D exam, along with a reimbursement code for general practitioners (GPs) to bill upon completing it. This study investigates whether GP adoption of the checklist improves adherence to recommended services for T2D patients.

**Methods:**

To investigate the impact of GP checklist adoption, we use Norwegian registry data from 2006 to 2021 and apply two difference-in-differences (DID) methods that account for staggered adoption timing: the two-way fixed effects (TWFE) estimator and Callaway and Sant’Anna’s DID method for variation in exposure timing (CSDID) (Callaway B. et al., J Econom 225:200–30, 2021).

**Results:**

We find that installing the electronic form has modest effects on the use of some recommended procedures.

**Conclusions:**

Our study suggests that using the electronic form can have a positive effect on recommended services. However, the modest impact indicates that installing the form does not necessarily translate into its active regular use.

**Supplementary Information:**

The online version contains supplementary material available at 10.1186/s12913-024-11940-x.

## Introduction

Type 2 diabetes (T2D) is a common and costly chronic condition that can lead to serious complications, including cardiovascular disease, blindness, chronic kidney disease, and amputations [1]. With its growing prevalence globally, T2D imposes a substantial societal burden, contributing to increased mortality, higher morbidity, reduced workforce participation, and rising healthcare costs.

Effective management of T2D by both patients and healthcare providers is critical for preventing or delaying its complications. In Norway, T2D care is primarily delivered through the primary care system, with general practitioners (GPs) playing a central role in patient management. Since 2001, Norwegian residents have had the option to register with a designated GP responsible for providing primary care and coordinating specialist referrals. During regular checkups, GPs educate patients on self-management, monitor risk factors and complications, prescribe medications, and refer complex cases to diabetes clinics or specialists. In 2020, an estimated 235,400 to 258,900 people in Norway were living with T2D [2].

The Norwegian Directorate of Health provides regularly updated evidence-based guidelines for the prevention, diagnosis, and treatment of diabetes, based on input from national medical experts [3]. These guidelines recommend that T2D patients attend regular checkups with their GP. For patients with poorly controlled diabetes or complications, shared care between GPs and specialists is advised to improve outcomes. To support these checkups, the Norwegian Organization for Quality Improvement of Laboratory Examinations (Noklus) introduced a diabetes form in 2008, designed for GPs to use during annual checkups for T2D patients [4]. This form, launched alongside the Norwegian Diabetes Register for Adults (NDR-A), serves as a checklist for systematically recording and monitoring clinical data, thereby enhancing the quality of diabetes care in Norway. GPs, outpatient specialists, and private endocrinologists are encouraged to submit patient data to the registry, linking electronic health records to the NDR-A through an integrated software system. Until recently, written patient consent was required for data submission.

The electronic form helps improve care by reminding GPs of the key areas that need to be assessed during annual checkups, followed by appropriate adjustments in treatment to meet clinical targets for key risk factors [3]. This form functions as a practical example of a “checklist,” as described by Gawande [5], which is especially useful in managing complex conditions requiring attention to multiple factors.

The annual checkup covers essential risk factors, including smoking status, body mass index, blood pressure, glycaemic control (HbA1c), lipid levels, renal function, and screening for complications such as neuropathy and retinopathy. Referrals to an ophthalmologist for eye exams are recommended every two years, even for patients without symptoms.

GPs are reimbursed for completing the checkup and submitting the form electronically using a billing code known as fee 109. This fee, introduced in 2008 at NOK 80 (approximately USD 8), was raised to NOK 200 (USD 20) by 2021 for the first annual checkup, and NOK 110 (USD 11) for subsequent checkups. Notably, there is no additional patient copayment beyond the standard consultation fee.

Our study examines whether the implementation of this diabetes form increases adherence to the procedures recommended by clinical guidelines. This is particularly important as many GPs do not consistently follow national guidelines. If the form proves effective in enhancing preventive care, it could serve as a cost-effective intervention. We use Norwegian registry data from 2006 to 2021 and apply a difference-in-differences (DID) approach to evaluate changes in the provision of recommended services for T2D patients after GPs adopt the form.

## Data sources, study sample, and measures

### Data sources

We use 2006–2021 data from the Norwegian GP registry and the Norwegian Control and Payment of Health Reimbursements Database (KUHR). The Norwegian GP registry contains demographic information on GPs and links GPs and registered patients. KUHR contains information on bills from health services, including type of services according to the fee schedule and relevant diagnoses.

### Outcomes

We use information from KUHR to construct outcome variables that indicate whether patients receive the recommended services. These services include electrocardiography, blood glucose testing, HbA1c testing, and microalbumin testing in general practice, as well as eye doctor visits in specialist care. The annual checkup aims to measure clinical risk factors, assess the risk of complications, and adjust relevant multifactorial treatment components. According to national diabetes guidelines, the HbA1c test is particularly important, as good blood sugar control is essential for effective diabetes management. The blood glucose test complements the HbA1c test, providing additional insights into achieving optimal glycaemic control. The microalbumin test and eye exams are important due to the elevated risk of diabetic nephropathy and retinopathy, respectively. Furthermore, the guidelines emphasize that patients with diabetes are at higher risk for cardiovascular disease. Electrocardiography should be performed upon diagnosing type 2 diabetes. Additionally, the Noklus form includes a question about whether the patient has atrial fibrillation, which must be confirmed through electrocardiography.

### Covariates

The Norwegian GP registry contains demographic information on GPs, including age, sex, and specialization in general practice. We use information from KUHR to calculate for each GP the number of registered patients who receive services for T2D in a year.

### Sample selection and descriptive statistics

The intervention is to adopt/install the Noklus form. Intervention adoption is staggered. Column 2 of Table 1 shows when GPs install the form. Our empirical strategy requires that we observe GPs before and after they install the form, therefore we drop GPs who install the form in the first year they practice. Column 3 of Table 1 shows the remaining GPs. For each GP and year, we keep registered patients with billing in KUHR with diagnosis code T90 (ICPC-2) or E11 (ICD-10) in that year (henceforth active T2D patients).

The earliest adopters installed the software in 2008. For the 2008-group, we have up to two years of pre-intervention data (2006–2007) and 14 years of post-intervention data (2008–2021). The latest adopters installed the software in 2021. For the 2021-group we have up to 15 years of pre-intervention data (2006–2020) and one year of post-intervention data (2021). GPs who never adopted can be observed for up to 16 years (2006–2021).

**Table 1:** Timing of adopting the checklist software

	Step 1: Raw data	Step 2: Drop GPs who do not have prior-adoption data	Step 3: Keep active T2D patients	
Adoption-timing	# GPs	# GPs	# GPs	# patients
2008	340	316	315	30 031
2009	641	563	563	51 423
2010	571	484	483	45 256
2011	531	421	417	37 312
2012	555	400	400	35 206
2013	635	457	457	41 771
2014	622	387	384	34 531
2015	538	323	320	27 950
2016	453	244	242	19 814
2017	437	212	210	14 987
2018	418	183	182	13 147
2019	409	163	158	11 269
2020	385	136	135	9 056
2021	345	124	124	7 400
Not adopted by Dec 31, 2021	2 291	2 291	2 266	93 096
Total	9 171	6 704	6 656	472 249

This table shows sample selection and adoption timing groups. Patients can switch GPs, so the number of unique patients in the sample is 340 252

In cases of staggered intervention adoption, DID compares GPs who have adopted the checklist with those who have not yet adopted it and those who never adopt it. The latter comparison may be less convincing because GPs who never adopt might be quite different. Thus, it is useful to investigate whether the never-adopted GPs are similar to adopted GPs. Table 2 compares the groups in 2007 baseline, when no one adopted the form. However, only 3 850 GPs (57% of the GPs in our sample) can be observed in 2007, because many new GPs started to practice each year after 2007. The two groups are not entirely comparable: the adopted GPs performed more recommended procedures even before they installed the software.

**Table 2:** Comparing 2007 baseline characteristics of adopting and never-adopting GPs

	Adopted	Never adopted
Number of GPs	2 864	966
Number of patients diagnosed with diabetes	87 314	25 057
Health service use among patients with diabetes		
% of patients who had electrocardiography	13*	12
% of patients who had blood glucose test	68***	63
% of patients who had HbA1c test	70***	64
% of patients who had microalbumin test	25***	19
% of patients who had an eye doctor visit	39*	40
GP characteristics		
Female (%)	32	30
Age group (years) (%)		
< 40	22	25
40–49	30***	22
50–59	38***	29
≥ 60	9***	23
Specialization in family medicine (%)	60***	45
Number of active T2D patients	30***	26

Two-sample t-tests were used to compare the means

*p < 0.10

***p < 0.01

## Empirical framework

We are investigating whether installing the Noklus diabetes form improves GPs’ adherence to recommended services. A key challenge in assessing this impact is the presence of confounding variables—GP characteristics that influence both their decision to install the form and their likelihood of performing recommended services. Table 2 illustrates that GPs who install the form differ from those who do not in terms of observable characteristics. Additionally, there are likely unobserved differences, such as GPs who install the form having higher professional standards or having patients requiring more follow-up. Fortunately, many of these characteristics are stable over time, allowing us to control for them using GP fixed effects.

However, using fixed effects alone to compare outcomes before and after form installation does not adjust for natural changes in outcomes over time. This comparison would thus reflect both the impact of form installation and underlying time trends. Therefore, to isolate the form’s effect, we compare changes in outcomes over time between GPs who installed the form and those who did not, accounting for both GP-specific differences (GP fixed effects) and common time trends (time fixed effects). Given panel data, a standard method for such comparisons is the two-way fixed effects (TWFE) regression model.

However, we must also consider the staggered adoption of the form since GPs install the software at different times. The TWFE estimator may then be biased if the intervention effect varies with the timing of installation [6]. It is plausible that the impact differs between GPs who adopted the form when it was first introduced in 2008 and those who implemented it a decade later. This variation is explored in the subgroup analysis presented in Table 4. An unbiased alternative is the CSDID estimator developed by Callaway and Sant’Anna [7]. Auxiliary analyses show that TWFE and CSDID estimators produce different effect estimates, indicating that TWFE produces potential bias.

Thus, we would like to use the CSDID estimator. A limitation of CSDID is its requirement of proper panel data with only one observation per GP per period. While patient-level data can be used with a CSDID repeated cross-section estimator, this approach sacrifices the GP fixed effects which are crucial for our analysis. Therefore, we aggregate the data to one observation per GP per year, which we believe does not result in significant information loss. In this aggregated dataset, we observe GPs over time, which enables us to use the CSDID panel data estimator. Firstly, the main source of potential bias is GP self-selection. Secondly, we are not interested in intervention effect heterogeneity between diabetes patients since national clinical guidelines recommend follow-up by the Noklus form for all diabetes patients. Thus, we focus on GP-level data to examine the effect of form installation on the *share* of patients receiving recommended services. This effect is measured by comparing GPs who have installed the form and GPs who have not installed the form using CSDID. This approach aims to identify the average treatment effect on the treated (ATT)—the effect of form installation among GPs who chose to implement it. For this comparison to identify the ATT, changes in shares among GPs who did not install the form must approximate those who did, had they not done so (the counterfactual). This requirement is known as the parallel trends assumption.

Specifically, we define a binary intervention variable, adopt, that equals one if the GP installed the Noklus diabetes form software and zero otherwise. Following Rubin (1974), the ATT is the difference in the average potential outcome for the intervention group if they adopted the form versus if they did not, where the latter is unobserved:

1 $$\:ATT=E\left[y_\text{post}^1\vert\text{adopt}=1\right]-E\left[y_\text{post}^0\vert\text{adopt}=1\right]$$

To estimate the ATT, we use the CSDID outcome regression estimator [7]. This estimator computes group-time ATTs, which are the intervention effects in period $\:t$ for GPs who adopted the form in period $\:g$, denoted as $\:ATT\left(g,t\right)$. To derive group-time ATTs, one keeps observations from periods $\:t$ (the intervention period) and $\:g-1$ (the baseline period) from GPs who adopted in period $\:g$ and those who had not yet adopted by period $\:t$. Then, using this subset of the data, one runs the following linear regression:

2 $$\:{Y}_{it}=\alpha\:+\mu\:{\text{Treat}}_{i}+\lambda\:{\text{Post}}_{t}+{\tau\:\left(\text{Treat*Post}\right)}_{it}+{\epsilon\:}_{it}$$

Here, $\:{Y}_{it}$ is the outcome for GP $\:i$ at time $\:t$, $\:\text{Treat}_{i}$ is an indicator variable that is equal to one for GPs in group $\:g$, $\:\text{Post}_{t}$ is an indicator that is equal to one at time $\:t$, and $\:{\epsilon\:}_{igt}$ is the residual term. The interaction term captures the intervention effect, $\:ATT\left(g,t\right)$. These group-time ATTs are then aggregated across post-intervention periods and timing groups to derive an overall ATT estimate. See Callaway and Sant’Anna [7] for details about the aggregation scheme.

**Fig. 1:**
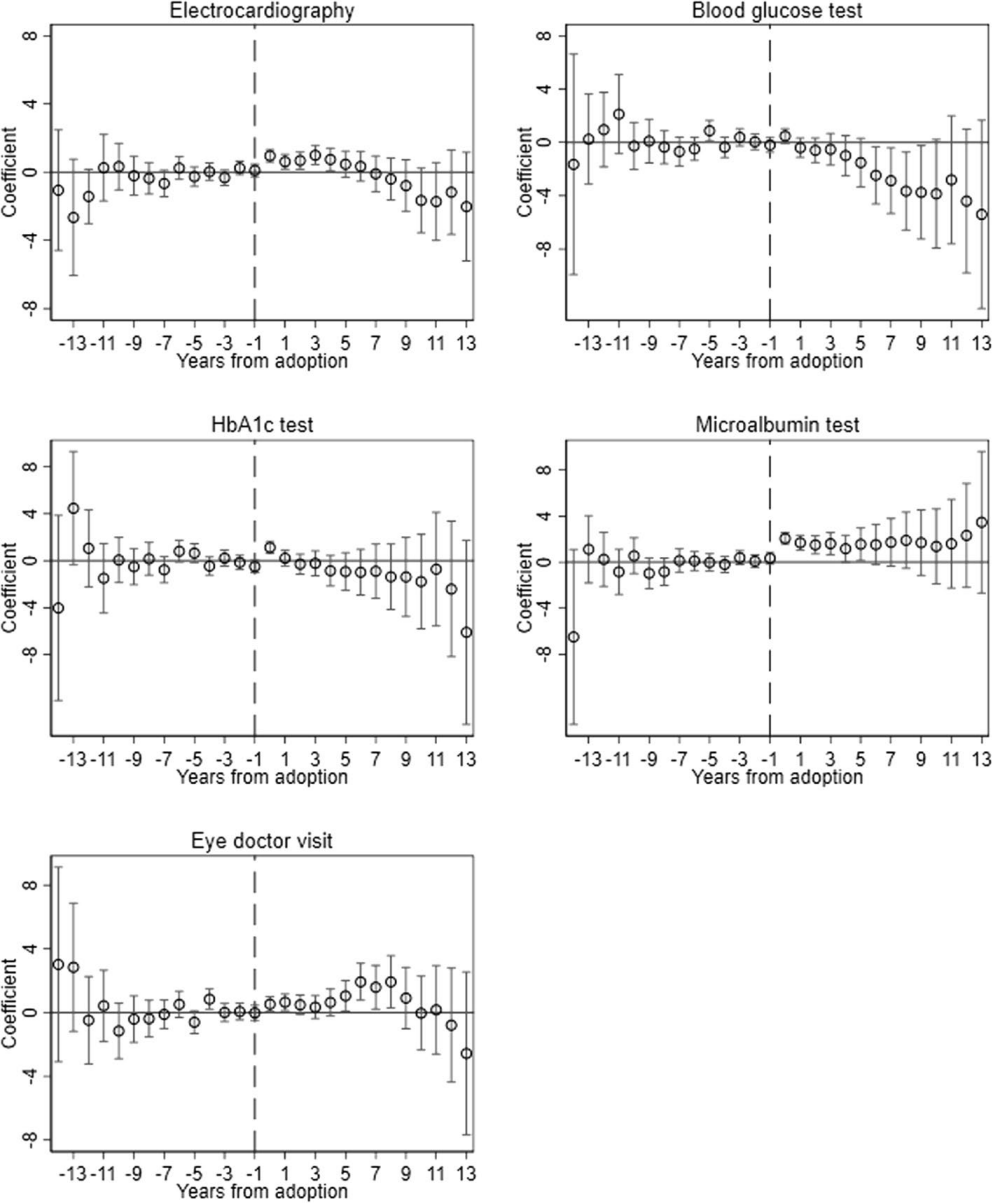
Event study effects of GPs adopting the checklist on recommended procedures Notes: Each panel shows estimates of the effect of installing the software by year from instalment using the DID method described in [7]. The capped spikes plot 95% confidence intervals constructed from standard errors clustered at the GP level

As mentioned above, our approach crucially relies on the parallel trends assumption. This assumption cannot be directly tested but it can be supported by observing parallel pre-adoption trends. To assess this, we analyse $\:ATT(g,t)$’s grouped by event-time (time from adoption). Reassuringly, event-time intervention effects reveal no effect prior to adoption (Fig. 1). Because we cannot definitively assert that trends would have remained parallel post-adoption (counterfactual), it is important to discuss whether it is likely. In doing so, one must think of the factors influencing changes in the share of patients receiving recommended services. One possibility is that installing the form may attract more proactive patients (patient self-selection). Nonetheless, we demonstrate that the effects remain largely unchanged when restricting analysis to patients who were on the GPs’ lists before form installation (Appendix Table A2 Panel A). Alternatively, intervention GPs might make different decisions compared to control GPs (aside from form installation), potentially leading to divergent trends (GP self-selection). A high professional standard may both initiate installation of the Noklus form and adoption of newly introduced medication. However, this concern is less impactful given our focus on immediate service changes rather than health outcomes. Targeted services are routine laboratory tests and referrals unlikely to have been initiated at the same time and independent of the installation of the Noklus form. Furthermore, the form’s effects are “direct” as it provides clear actions for GPs to follow. If we still have concerns about the parallel trends assumption, the staggered design allows flexibility in choosing controls—whether from GPs who never installed the form, those who have not yet installed it, or both. Again, we show that our estimates are robust across these choices. Therefore, we prefer to include both groups, which enhances the precision of our findings due to the larger sample size.

In addition to parallel trends, identification requires that the intervention is absorbing, meaning once GPs install the software, they cannot uninstall it. Additionally, GPs should not change their behaviour in anticipation of the intervention. It is reasonable to rule out anticipatory effects. One might think that GPs would delay checkups until after installing the software to claim fee 109. However, this concern does not affect our estimates because the fee can be claimed yearly, and our data is aggregated annually. Moreover, GPs are not required to complete the checkup and fill in the form on the same day. The absorbing intervention assumption implies that GPs cannot uninstall the software. However, GPs who end up not using the form might do so, especially those who because of their electronic health record system face an annual licensing fee. If GPs uninstall the software, our estimates might underestimate the true intervention effect.

To sum up: The intervention is endogenous since GPs themselves choose whether to install the Noklus form. Endogeneity is a potential threat to effect estimation since high professional standard may both initiate installation of the Noklus form and other innovations (GP self-selection). Also, pro-active patients may be attracted to GPs with a high professional standard (patient self-selection). We have argued that neither GP self-selection nor patient self-selection are likely to represent a threat to estimating the effect of the Noklus form in the present study.

To strengthen our confidence in the validity of our findings, we conduct subgroup analyses and perform several robustness checks. In the subgroup analysis, we divide the sample into early, middle, and late adopters to assess whether the effects of adopting the form have changed as its use became more common. As part of the robustness checks, we examine whether the results are influenced by factors such as the adoption of the form attracting new patients, the impact of the COVID-19 pandemic, and our decision to aggregate the data to utilize the CSDID panel data estimator.

## Results

Table 3 shows the mean effects of the GP adopting the checklist. Specifically, adopting the checklist increases proportions of patients with annual microalbumin tests by 1.6 ppt. and annual eye doctor visits by 0.7 ppt. This represents relative increases of 7.5% and 1.8% from the baseline, respectively. There were no effects on electrocardiography, blood glucose test and HbA1c test.

Figure 1 plots event study effects over years from adoption. The figure shows that the increase in annual microalbumin tests is immediate and quite stable. The effect on eye doctor visit gradually increases initially and then gradually decreases over time. The decrease in 2020–2021 is likely due to COVID-19 pandemic effects. On all outcomes, there is a significant increase in the year when the GP first adopted the checklist.

Table 4 shows the results by early adopters, middle adopters, and late adopters. The corresponding event study results are shown in Appendix Figure A1. This subgroup analysis is relevant because only the early cohort was exposed for an extended period, and it is possible that effects may become easier to detect after some time has passed. We do not observe a consistent dose relationship between timing of adoption and magnitude of effects. The middle adopters have the largest increase in the probability of electrocardiography, while the late adopters have the largest increase in the probability of an eye doctor visit.

**Table 3:** Overall effects of GPs’ adopting the checklist on recommended procedures

	Baseline	ATT (SE)	Percent change from baseline
Electrocardiography	13.5	0.361 (0.302)	2.6
Blood glucose test	56.1	−1.338 (0.688)	−3.4
HbA1c test	70.5	−0.420 (0.659)	−0.5
Microalbumin test	22.0	1.666** (0.564)	7.5
Eye doctor visit	42.6	0.773* (0.391)	1.8

Baseline is the average outcome value in the intervention group in period − 1. ATT is the overall effect of installing the software, i.e., the average of the post-intervention event study effects. The effects are estimated using the DID method described in [7]. The standard errors are clustered at the GP level. *N* = 57 703 is the number of GPs x years

*p < 0.05

**p < 0.01

**Table 4:** Effects of GPs’ adopting the checklist on recommended procedures by GPs’ adoption time

	Baseline	ATT (SE)	Percent change from baseline
Early adopters (*N* = 32 730)			
Electrocardiography	13.9	−0.160 (0.529)	−1.1
Blood glucose test	63.8	−1.085 (1.155)	−1.6
HbA1c test	70.6	−0.360 (1.228)	−0.5
Microalbumin test	26.4	2.024* (0.967)	7.6
Eye doctor visit	43.1	0.405 (0.677)	0.9
Middle adopters (*N* = 25 993)			
Electrocardiography	13.1	0.791* (0.386)	6.0
Blood glucose test	52.1	−0.592 (0.971)	−1.1
HbA1c test	70.7	1.157 (0.849)	1.6
Microalbumin test	18.2	2.433*** (0.738)	13.3
Eye doctor visit	42.2	1.391* (0.553)	3.2
Late adopters (*N* = 13 559)			
Electrocardiography	13.7	0.732 (0.590)	5.3
Blood glucose test	38.9	−0.770 (0.924)	−1.9
HbA1c test	69.5	−0.011 (0.889)	−0.0
Microalbumin test	15.7	1.463 (0.844)	9.3
Eye doctor visit	41.7	2.097** (0.752)	5.0

Baseline is the average outcome value in the intervention group in period − 1. ATT is the overall effect of installing the software, i.e., the average of the post-intervention event study effects. The effects are estimated using the DID method described in [7]. The standard errors are clustered at the GP level. Early installed the software in 2008–2012, middle installed the software in 2013–2017, late installed the software in 2018–2021. N is the number of GPs x years

*p < 0.05

**p < 0.01

***p < 0.001

We perform several robustness checks to confirm the results. First, we limit the sample to patients who stayed with the same GP before and after the GP adopted the checklist, ensuring the results aren’t influenced by new patients seeking more procedures. The findings remain consistent. Second, we exclude the COVID-19 pandemic years, which may have disproportionately affected preventive services. The results still hold, with a small positive effect on electrocardiography. Third, a placebo test on C-reactive protein (CRP) tests, not included in the checklist, shows no effect. We also use a TWFE DID model, which assumes constant intervention effects and parallel trends—assumptions not fully met by the data. While the results are similar to the main analysis, intervention effects vary over time, and event studies show non-parallel pre-trends, supporting our preference for the CSDID estimator. Finally, when we rerun the analysis on GPs who actively used the checklist at least once per year, the effects are larger, indicating that regular use of the form increases its impact. Details and results from the robustness checks are in Appendix.

## Conclusions

 The aim of the Noklus diabetes form is to aid follow-up of T2D patients by reminding GPs about what should be assessed at an annual checkup. We find that installing the Noklus diabetes form helps remind GPs to undertake some important recommended procedures particularly regarding microvascular complications (eye, kidney disease) that are often forgotten without the form. However, the effects are small. The main reason for small effects is likely that forms are not consistently used for all patients after they were installed. Appendix Table A1 shows that many GPs who adopt the form rarely use it for follow-up of active T2D patients, and that there is large variation in usage. In the year that the GPs installed the software, 384 GPs did not use the form on any active T2D patients, 2 044 GPs used it on one active T2D patient, and 16 GPs on at least 40 active T2D patients. 75% of adopting GPs had at least 19 active T2D patients in the year before they installed the software. On the other procedures, electrocardiography, blood glucose test and HbA1c test, there seem to be an initial increase following the adoption, but the effect does not last. This could be due to GPs “always” measuring HbA1c for their patients, so there is no need to be reminded to do this. Policymakers have recommended that GPs adopt the form. According to Table 1, 65% of the GPs have adopted the form during our observation period. Hence, the recommendation has been quite successful. Bakke et al. [8] report that only 26% of GPs in their sample actually use the Noklus diabetes form. They find that use of the form was associated with improved screening of T2D patients. Hence, their results correspond to our results displayed in Appendix Table A2 Panel E showing a key distinction between simply installing the form and actually using it. Additional incentives, for instance in terms of increased fees, to make GPs use the form could be considered.

Our findings suggest that the checklist works partially as intended: GPs who installed the form are more likely to perform some important recommended procedures. This result is consistent with what Nøkleby [9] found, that the use of the form was strongly associated with the number of recommended procedures performed by the GP. Our results are also consistent with the examples that Gawande [5] referenced for the airline industry and in health care. Another example was reported in Haynes et al. [10], who examined hospitals that participated in the World Health Organization’s Safe Surgery Saves Lives program. The rate of complications, including death, during hospitalization within the first 30 days after the operation were reported before and after the introduction of the Surgical Safety Checklist. Authors found that both inpatient complications and mortality reduced after the checklist’s introduction.

Only a small body of literature has investigated the use of structured forms on preventing complications. Larun et al. [11] reviewed seven randomized controlled trials comparing adults with T2D who had follow-up in a primary health care setting with and without forms in some western countries. Previous research has lacked longitudinal data to measure health outcomes such as mortality and other complications. Only one study included primary outcomes such as mortality and incidence of myocardial infarction. Some promising effects have been found on secondary outcomes, such as HbA1c and blood pressure, although the magnitudes are small. The authors concluded that published data provided no clear answers, calling for an empirical assessment of the benefits of employing the Noklus diabetes form. A follow-up of the present study would be to study the Noklus form’s effect on disease complications and hospitalizations.

Our study design has several potential limitations. We examine the effect of installing the software. The instalment is a condition for doing the annual checkup that authorizes the claim of fee 109. The instalment is an investment decision, as analysed by Iversen and Ma [12]. The software is offered free of charge by Noklus. Hence, the investment cost consists of the time cost involved in installing and integrating the software with patient electronic record. It also involves entering baseline data and learning to use the software. The time involved has an opportunity cost in terms of fee generating patient consultations. In two geographical areas, Noklus offered the GPs assistance from a diabetes nurse with installing and learning to use the software. These areas experienced an increase in installation of the software compared with other areas. Hence, time costs are considered by GPs when making the investment decision. The investment is implemented if the present value of benefits exceeds the present value of costs. For instance, young GPs with many T2D patients can benefit from the investment in more years than older GPs with few T2D patients and are thus more likely to invest. At first sight, it seems surprising that a considerable number of GPs who have installed the software but do not consistently use the Noklus diabetes form. One reason can be that the opportunity cost of using the form has developed differently from what was expected at the time the investment was made. The first comprehensive checkup with the Noklus form may be time-consuming because more information about patient characteristics are entered the first time, but other health personnel (nurses/medical secretaries) might assist with this. Perhaps some T2D patients have other pressing issues to discuss and require more time than was expected. Subsequent 109 fee is much less than the first time with a new patient, so it may not be worthwhile for GPs to fill in the form each time.

We use information from KUHR to construct outcome variables that indicate whether patients receive the recommended services. The registration has two limitations. First, we only register services that have a fee that GPs or physician specialists are entitled to. Since we only register some of the relevant procedures, our study design is conservative. Even if we do not have access to all relevant procedures, we should still be able to conclude whether installing the checklist impacts the use of recommended procedures. The second limitation is that we only capture laboratory analyses performed at the GP office. Some GPs may collect blood samples and send them to an external laboratory for analysis. However, the tests we include—blood glucose, HbA1c, and microalbumin—are all recommended to be analysed at the GP office [13].

The main analysis implicitly assumes that the investment decision is exogenous to patients. This means that patients are neither involved in the investment decision nor are more likely to switch to GPs that are more likely to install the software or have recently installed the software. We know from the literature that patients may consider quality indicators when they choose the GP to be listed with. With data from England, Santos et al. [14] find that individuals are more likely to choose practices of higher quality as measured by publicly available data on practice performance. Mokienko and Wangen [15] find that chronic patients who use primary care intensively are less likely to disenroll GPs who have a high share of patients with the same diagnosis. Applied to our topic, this means that patients with T2D disenroll less often from GPs who care for many patients with T2D. In Appendix Table A2 Panel A, we presented the results from an analysis with the subpopulation of patients who were registered with the same GP both before and after the adoption. The results were similar to our main results. We conclude that patient endogeneity is not likely to be a problem.

Regarding GP selection, Table 2 shows that there was a difference between adopting and non-adopting GPs before the adoption of the diabetes form. It seems that adopting GPs do more of the recommended procedures even before they install the software. This has two implications. First, differences in levels of outcomes need not be a threat to the validity of our results. Instead, we cannot have differences in trends of outcomes. Our event study shows pre-trends are very similar across groups (Fig. 1). In addition, GP practice style likely does not vary much over time [16]. Second, the fact that GPs who never installed the form perform fewer of the recommended procedures suggests they could gain more from adopting the form compared to those who have already implemented it. This tells us that in addition to the importance of making GPs who install the form also use it, it may be useful to continue recruiting non-adopting GPs.

Finally, our study design requires that we assume that GPs do not uninstall the software. If GPs in fact do uninstall the software, we underestimate the intervention effect estimates. Furthermore, we require that GPs are observed for at least one year before they install the software, i.e., we drop GPs who adopt the form the same year they start practicing (see step 2 in Table 1). We observe that the form becomes more standard over time, thus our results may be less generalizable to GPs who newly started to practice.

During recent years, new drug classes for the treatment of T2D have appeared. GLP-1 agonists and SGLT2 inhibitors have so far been successful in improving health outcomes and reduce the risk of disease complications in patients with T2D [17]. The new drug classes are included in the Norwegian guidelines from the Directorate of Health. Improved treatment options may encourage physicians to monitor their T2D patients more regularly to identify candidates for the improved treatment, which may affect physicians’ behaviour on following guidelines. However, it is unclear whether improved follow-up encourages the installation of the electronic form.

Some Norwegian GPs think that the Noklus form is not useful because GPs already provide the recommended services [18]. Our study has shown that the Noklus form does increase recommended services, contributing to the literature demonstrating that checklists matter. The criticism is more common among GPs who choose not to adopt the form. We estimate the average intervention effect on the adopters. From Table 2, we see that the GPs who choose to never adopt are less likely to provide recommended services without the form than GPs who choose to adopt the form. Hence, the potential for improved practice is greater among the critics than for GPs who adopt the Noklus form.

## Supplementary Information

Supplementary Material 1.

## Data Availability

Access to the data used in the study can be obtained from the Norwegian GP Registry (https://helsedata.no/en/forvaltere/norwegian-directorate-of-health/norwegian-gp-registry/) and the Norwegian Control and Payment of Health Reimbursements Database (https://helsedata.no/en/forvaltere/norwegian-directorate-of-health/norwegian-control-and-payment-of-health-reimbursements-database-kuhr/). The process for data access and linkage involves obtaining ethical approval, permission to process personally identifiable information, and an exemption from confidentiality agreements for each data source.

## Abbreviations

T2D: Type 2 diabetes
GP: General practitioner
DID: Difference-in-differences
Noklus: Norwegian Organization for Quality Improvement of Laboratory Examinations
NDR-A: Norwegian Diabetes Register for Adults
KUHR: Norwegian Control and Payment of Health Reimbursements Database
TWFE: Two-way fixed effects
ATT: Average treatment effect on the treated
CRP: C-reactive protein
