# Supplementary material for: Effects of a national checklist on recommended procedures among patients with type 2 diabetes
Source: BMC Health Serv Res. 2024 Nov 26;24:1472. doi: 10.1186/s12913-024-11940-x (PMC11590329; doi:10.1186/s12913-024-11940-x)
Supplement: Supplementary file 1 — Supplementary Material 1. [file 12913_2024_11940_MOESM1_ESM.docx]

**Appendix**

**Table A1** Number of intervention GPs who provide follow-up with the use of the Noklus diabetes form to 0, 1, 2-4, 5-9… active T2D patients 0-13 years after installing the form

| Event time | # GPs with 0 checklists | # GPs with 1 checklist | 2-4 | 5-9 | 10-19 | 20-39 | 40+ | Total |
| --- | --- | --- | --- | --- | --- | --- | --- | --- |
| 0 | 384 | 2 044 | 1 099 | 474 | 256 | 117 | 16 | 4 390 |
| 1 | 1 800 | 608 | 540 | 380 | 286 | 147 | 39 | 3 797 |
| 2 | 1 670 | 508 | 474 | 320 | 246 | 139 | 54 | 3 411 |
| 3 | 1 519 | 474 | 393 | 238 | 248 | 146 | 64 | 3 082 |
| 4 | 1 291 | 378 | 361 | 264 | 243 | 141 | 74 | 2 752 |
| 5 | 1 104 | 336 | 331 | 230 | 243 | 136 | 72 | 2 452 |
| 6 | 977 | 267 | 264 | 219 | 208 | 148 | 59 | 2 142 |
| 7 | 869 | 205 | 207 | 188 | 185 | 126 | 68 | 1 848 |
| 8 | 710 | 179 | 171 | 136 | 154 | 111 | 65 | 1 526 |
| 9 | 545 | 141 | 132 | 109 | 124 | 109 | 48 | 1 208 |
| 10 | 454 | 98 | 87 | 85 | 94 | 90 | 46 | 954 |
| 11 | 327 | 83 | 70 | 43 | 72 | 77 | 36 | 708 |
| 12 | 216 | 44 | 33 | 34 | 39 | 59 | 16 | 441 |
| 13 | 71 | 16 | 11 | 11 | 24 | 19 | 7 | 159 |

Notes: This table shows counts of intervention GPs based on the number of active T2D patients who receive follow-up with the use of the Noklus diabetes form. GPs may also use the form for follow-up of patients other than active T2D patients (mainly T1D patients, but also “inactive” T2D patients, i.e., T2D patients without bill in KUHR with diagnosis code T90 (ICPC-2) or E11 (ICD-10) in the particular year, or patients who are not registered with the GP in the particular year (if a patient registers after 1 January, they are not considered registered until next year).

**Fig****ure A1** Event study effects of GP adopting the checklist on recommended procedures by adoption timing


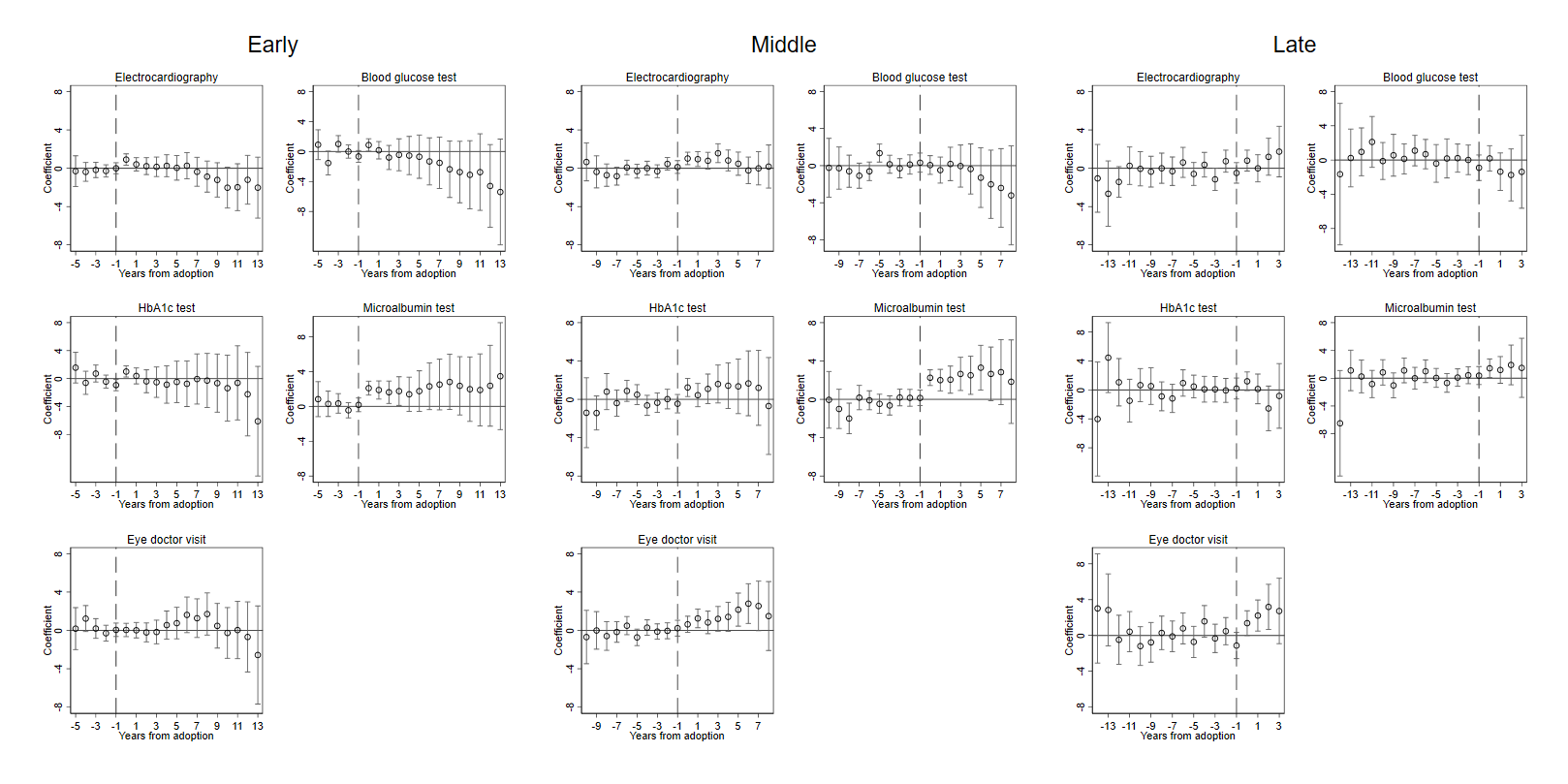


Notes: Each panel shows estimates of the effect of installing the software by year from instalment using the DID method described in [2]. The capped spikes plot 95% confidence intervals constructed from standard errors clustered at the GP level.

**Robustness analysis**

We conduct several robustness checks and their corresponding event study. Results from robustness checks are in Table A2 and figures to show results from event studies are in Figure A2-A5. First, we constrain the sample to patients who registered with the same GPs before and after GP adopted the checklist. This exercise is motivated by the concern that adopted GPs may attract patients who demand more recommended procedures, which would lead to overestimating the intervention effects. It is reassuring to find that the results are similar to the main results (Table A2 Panel A).

Second, we exclude the years with prevalent COVID-19 pandemic. Even if the pandemic affected everyone, its effects may be especially large on preventive services like annual diabetes checkups. Again, the results are similar to the main results (Table A2 Panel B), and now we see a small positive effect on electrocardiography.

Third, we conduct a placebo test to test the effect of checklist on the proportion of patients receiving C-reactive protein (CRP) blood test that checks for inflammation in the body. CRP test is not a procedure included in the checklist (Table A2 Panel C), and we don’t see any effects.

Fourth, we estimate a two-way fixed effect (TWFE) DID, which is commonly used for staggered adoption.

Eq. 3

$$y_{ijt} = \mu_{j}+\lambda_{t}+\theta\text{adopt}_{jt}+x_{ijt}\beta+\varepsilon_{ijt},$$

with corresponding event study equation:

Eq. 4

$$y_{ijt} = \mu_{j}+\lambda_{t}+\sum_{r = -15, r \neq-1}^{r = 13} \theta_{r}I_{jt}+x_{ijt}\beta+\varepsilon_{ijt}.$$

$y_{ijt}$ is the outcome for patient $i$ with GP $j$ in year $t$, $\mu_{j}$ represents GP fixed effects, $\lambda_{t}$ denotes calendar year fixed effects, and $x_{jit}$ is a vector of covariates. In Eq. 3, $\text{adopt}_{jt}$ is an indicator that equals one after the GP adopted the form. In Eq. 4, $\text{adopt}_{jt}$ is replaced by a set of indicators, $I_{r}$, for year from adoption. Thus, $\theta$ in Eq. 3 seeks to capture the overall effect of the form, while $\theta_{r}$ in Eq. 4 seeks to capture the effect of the form $r$ years from adoption.

Unlike the CSDID estimator, the TWFE estimator is conducted at the patient level data, which allows us to better control for patient characteristics. It produces interpretable coefficients on covariates; and it is familiar to many readers. However, Goodman-Bacon [3] shows that a causal interpretation of TWFE DID estimates requires both a parallel trends assumption and intervention effects that are constant over time. **Figure 1** shows that intervention effects are not constant over time. Table A2 Panel D shows results from estimating Eq. 3. We note that the significant effects in the main analyses are also significant here. It is also interesting to note that the effects on electrocardiography and HbA1c test are significant. Figure A4 shows estimates for $\hat{\theta}_{r}$’s from Eq. 4. The TWFE event studies do not suggest parallel pre-trends. Abraham and Sun [1] show that intervention effect heterogeneity can cause pre-trends to falsely arise. So, our preferred specification is CSDID as shown in our main results.

Finally, Table A2 Panel E shows the result of rerunning the analysis on GPs who use the form at least once per year (on active T2D patients) since installing the software. These results are a lot larger. This strengthens our suspicion that there is a key distinction between simply installing the form and using it.

**Table A2** Overall effects of GP adopting the checklist

|  | Baseline | ATT (SE) | Percent change from baseline |
| --- | --- | --- | --- |
| Panel A. Same patients registered with GPs before and after (N = 57 510) | | | |
| Electrocardiography | 13.4 | 0.265 (0.314) | 1.9 |
| Blood glucose test | 55.9 | -0.973 (0.708) | -1.7 |
| HbA1c test | 70.3 | -0.147 (0.691) | -0.2 |
| Microalbumin test | 22.0 | 2.136*** (0.585) | 9.7 |
| Eye doctor visit | 43.0 | 0.753 (0.410) | 1.7 |
| Panel B. Excluding COVID period 2020-2021 (N = 51 129) | | | |
| Electrocardiography | 13.6 | 0.583* (0.277) | 4.2 |
| Blood glucose test | 56.9 | -1.168 (0.627) | -2.0 |
| HbA1c test | 70.6 | -0.431 (0.583) | -0.6 |
| Microalbumin test | 22.2 | 1.45** (0.502) | 6.5 |
| Eye doctor visit | 42.6 | 0.702* (0.348) | 1.6 |
| Panel C. Placebo test (N = 57 703) | | | |
| CRP test | 34.2 | -0.401 (0.368) | -1.1 |
| Panel D. Two-way fixed effects estimates (N = 2 131 400) | | | |
| Electrocardiography | 13.7 | 0.889*** (0.178) | 6.5 |
| Blood glucose test | 59.7 | 0.233 (0.418) | 0.4 |
| HbA1c test | 73.6 | 0.905* (0.376) | 1.2 |
| Microalbumin test | 23.8 | 2.086*** (0.348) | 8.8 |
| Eye doctor visit | 41.9 | 0.965*** (0.205) | 2.3 |
| Panel E. GPs who use the form (N = 19 826) | | | |
| Electrocardiography | 14.2 | 2.030*** (0.477) | 14.2 |
| Blood glucose test | 54.5 | -1.364 (1.019) | -2.5 |
| HbA1c test | 70.4 | 1.211 (0.964) | 1.7 |
| Microalbumin test | 21.5 | 5.618*** (0.925) | 26.0 |
| Eye doctor visit | 42.9 | 2.112*** (0.615) | 4.9 |

Notes: Baseline is the average outcome value in the intervention group in period -1. ATT is the overall effect of installing the software, i.e., the average of the post-intervention event study effects. In Panels A, B, C and E, the effects are estimated using the DID method described in [2], and N is the number of GPs x years. In Panel D, the effects are estimates for θ from Eq. 3, and N is the number of patients x years. In all panels, the standard errors are clustered at the GP level. * p<0.05, ** p<0.01, *** p<0.001.

**Figure A2** Event study effects of GP adopting the checklist on recommended procedures. Same patients registered with GPs before and after


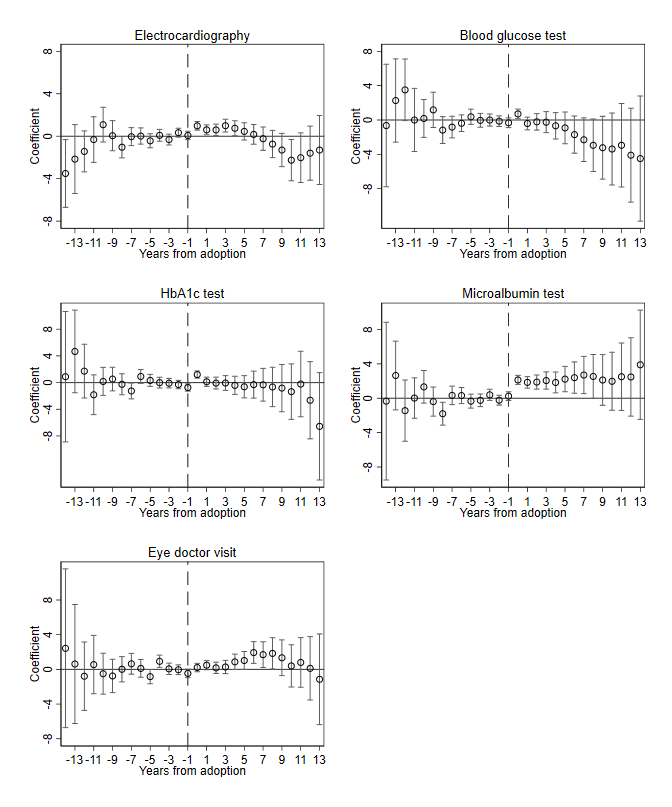


Notes: Each panel shows estimates of the effect of installing the software by year from instalment using the DID method described in [2]. The capped spikes plot 95% confidence intervals constructed from standard errors clustered at the GP level.

**Figure A3** Event study effects of GP adopting the checklist on recommended procedures. Excluding COVID period 2020-2021


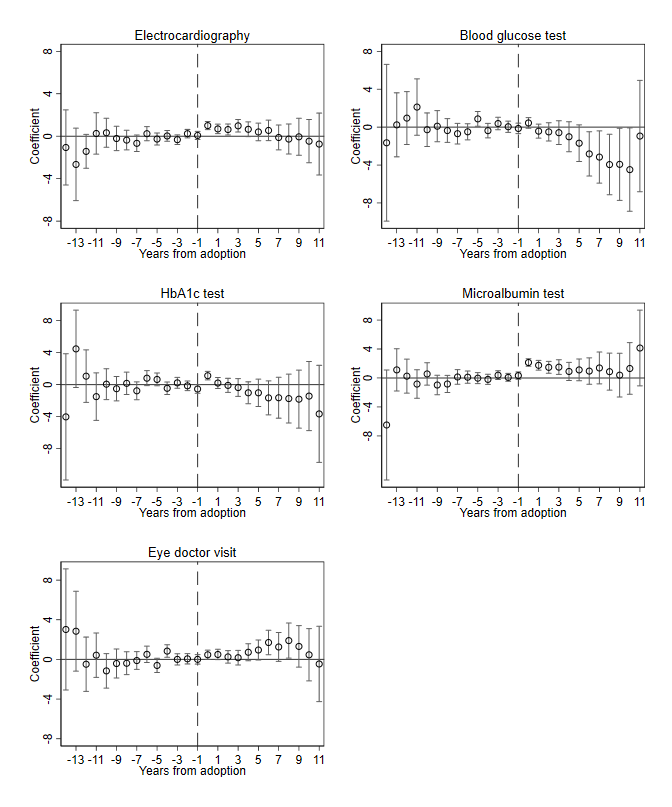


Notes: Each panel shows estimates of the effect of installing the software by year from instalment using the DID method described in [2]. The capped spikes plot 95% confidence intervals constructed from standard errors clustered at the GP level.

**Figure A4** Event study effects of GP adopting the checklist on recommended procedures. Two-way fixed effects estimates


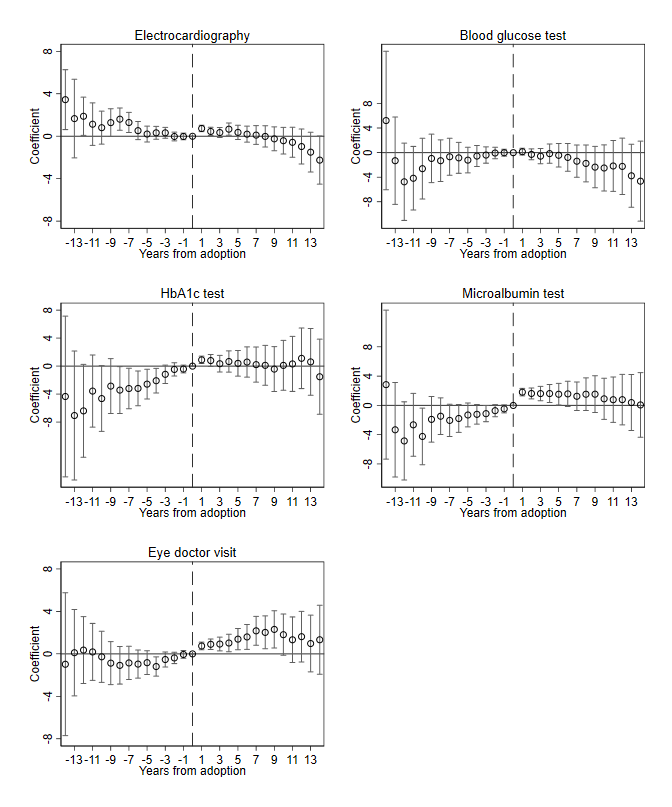


Notes: Each panel shows estimates of the effect of installing the software by year from instalment, i.e., the estimates for $\hat{\theta}_{r}$’s from Eq. 4. The capped spikes plot 95% confidence intervals constructed from standard errors clustered at the GP level.

**Figure A5** Event study effects of GP adopting the checklist on recommended procedures. GPs who use the form


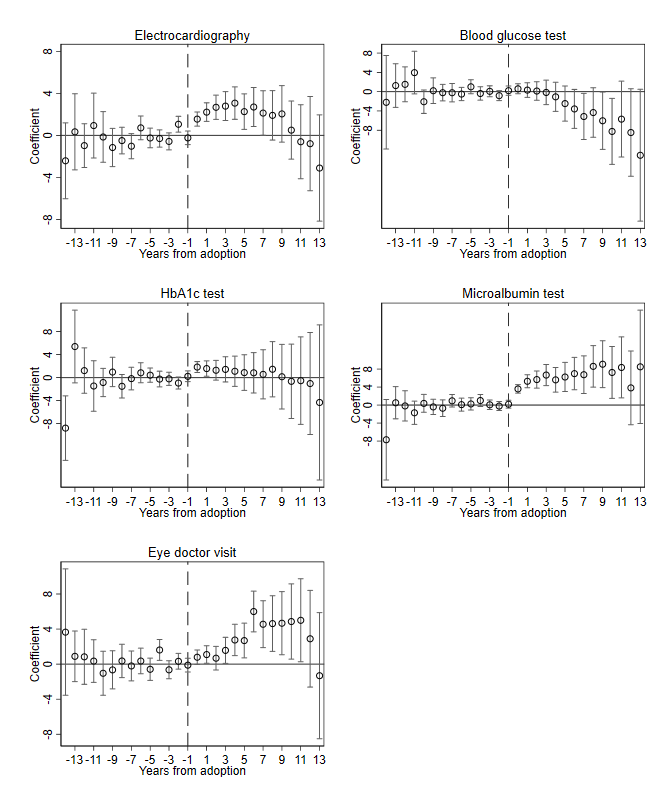


Notes: Each panel shows estimates of the effect of installing the software by year from instalment using the DID method described in [2]. The capped spikes plot 95% confidence intervals constructed from standard errors clustered at the GP level.

**References**

1. Abraham S, Sun L. Estimating dynamic treatment effects in event studies with heterogeneous treatment effects. J. Econom. 2021;225(2):175–199. https://doi.org/10.1016/j.jeconom.2020.09.006

2. Callaway B, Sant’Anna PHC. Difference-in-differences with multiple time periods J. Econom. 2021;225(2):200–230. https://doi.org/10.1016/j.jeconom.2020.12.001

3. Goodman-Bacon A. Difference-in-differences with variation in treatment timing. J. Econom. 2021;225(2):254–277. https://doi.org/10.1016/j.jeconom.2021.03.014
